# Blood Feeding and Insulin-like Peptide 3 Stimulate Proliferation of Hemocytes in the Mosquito *Aedes aegypti*

**DOI:** 10.1371/journal.ppat.1002274

**Published:** 2011-10-06

**Authors:** Julio Castillo, Mark R. Brown, Michael R. Strand

**Affiliations:** 1 Department of Entomology, University of Georgia, Athens, Georgia, United States of America; 2 Center for Tropical and Emerging Global Diseases, University of Georgia, Athens, Georgia, United States of America; Stanford University, United States of America

## Abstract

All vector mosquito species must feed on the blood of a vertebrate host to produce eggs. Multiple cycles of blood feeding also promote frequent contacts with hosts, which enhance the risk of exposure to infectious agents and disease transmission. Blood feeding triggers the release of insulin-like peptides (ILPs) from the brain of the mosquito *Aedes aegypti*, which regulate blood meal digestion and egg formation. In turn, hemocytes serve as the most important constitutive defense in mosquitoes against pathogens that enter the hemocoel. Prior studies indicated that blood feeding stimulates hemocytes to increase in abundance, but how this increase in abundance is regulated is unknown. Here, we determined that phagocytic granulocytes and oenocytoids express the *A. aegypti* insulin receptor (*AaMIR*). We then showed that: 1) decapitation of mosquitoes after blood feeding inhibited hemocyte proliferation, 2) a single dose of insulin-like peptide 3 (ILP3) sufficient to stimulate egg production rescued proliferation, and 3) knockdown of the *AaMIR* inhibited ILP3 rescue activity. Infection studies indicated that increased hemocyte abundance enhanced clearance of the bacterium *Escherichia coli* at lower levels of infection. Surprisingly, however, non-blood fed females better survived intermediate and high levels of *E. coli* infection than blood fed females. Taken together, our results reveal a previously unrecognized role for the insulin signaling pathway in regulating hemocyte proliferation. Our results also indicate that blood feeding enhances resistance to *E. coli* at lower levels of infection but reduces tolerance at higher levels of infection.

## Author Summary

Mosquitoes are vectors of several important diseases of humans and other mammals including Dengue fever, malaria and filariasis. These diseases adversely affect worldwide health by killing or debilitating millions of individuals. The key feature of mosquito biology that makes them such important disease vectors is that adult females must feed on the blood of their vertebrate host(s) to produce eggs. In turn, repeated bouts of blood feeding and egg development elevate the risk of mosquitoes feeding on an infected host and transmitting a given pathogen from one individual to another. A key regulator of egg development following blood feeding is the release of insulin-like peptides from the mosquito brain. We have found that insulin-like peptides enhance production of immune cells (hemocytes) that serve as the first line of defense against infection. Conversely, the molecular pathways that regulate egg development and hemocyte proliferation reduce the ability of mosquitoes to tolerate a persistent systemic infection. Taken together, our results indicate that trade-offs exist between reproduction and immune defense in mosquitoes, which is a subject of fundamental interest to evolutionary biologists and of applied importance in understanding disease transmission.

## Introduction

Insects primarily rely on an innate immune system for defense against pathogens and other foreign invaders. Reciprocally, a number of insects including mosquitoes can be infected by and transmit certain pathogens that cause disease in humans and other vertebrate hosts. The key feature of mosquito biology that makes them important disease vectors is that adult females must feed on the blood of their vertebrate host(s) to produce eggs. Repeated bouts of blood feeding and egg development in turn increases the risk of mosquitoes feeding on an infected host and transmitting a given pathogen from one individual to another.

At the physiological level, blood feeding by the mosquito *Aedes aegypti* triggers neurosecretory cells in the brain to release insulin-like peptides (ILPs) into circulation, which stimulate the ovaries to secrete ecdysteroid hormones (ECDs) [1]–[3]. ILPs from the brain together with nutrient sensing through the target of rapamycin (TOR) pathway also induce the midgut to produce enzymes that digest the protein-rich blood meal into amino acids. These amino acids are then transported from the midgut into the hemolymph where they are taken up by the fat body [4]–[6]. Amino acids, ECDs and ILPs together induce the fat body to produce vitellogenin (Vg) and other yolk proteins (YP) [7], [8], which are packaged into eggs that the female lays between 72 and 96 h post-blood meal (pbm). Pathogens in a blood meal infect mosquitoes during this same period by moving across the midgut, disseminating through the hemocoel, and invading other organs like the salivary glands or fat body.

Constitutive immune defenses in insects refer to factors like hemocytes that are always present and ready to act if an organism is infected, while inducible defenses refer to molecules like antimicrobial peptides that are produced in response to infection [9]. In most insects including mosquitoes, hemocytes serve as the most important constitutive defense element against pathogens that enter the hemocoel [10], [11]. Key functions of mosquito hemocytes include phagocytic and melanotic immune responses [12]–[17], production of soluble effector molecules with activity against bacteria and protozoa [17]–[23], and enhanced defense associated with immune priming [24]. *A. aegypti* produces three types of hemocytes named granulocytes, oenocytoids, and prohemocytes that are distinguished from one another by morphological, antigenic and functional characters [15]. In adult females, granulocytes account for more than 90% of the total hemocyte population and are the only cell type that is phagocytic. Oenocytoids in contrast are weakly adhesive cells that constitutively express components of the phenoloxidase cascade that regulate the formation of melanin, while prohemocytes are non-adhesive and may be putative progenitor cells.

Prior studies indicate that the number of hemocytes in adult *A. aegypti* progressively declines with age, which in turn correlates with age-dependent increases in susceptibility to infection by bacteria like *Escherichia coli* [13], [15]. In contrast, blood feeding stimulates an increase in hemocyte abundance in adult females that coincides with the timing of yolk deposition into follicles to produce eggs [15], [17]. In the current study, we hypothesized that circulatory hormones that stimulate egg development after blood feeding also mediate increases in hemocyte abundance. Our results show that loss of signaling from the mosquito brain after blood feeding inhibits the increase in hemocyte abundance that occurs after a blood meal while a single dose of insulin peptide 3 (ILP3), sufficient to stimulate egg development, rescues hemocyte proliferation.

## Materials and Methods

### Ethics statement

This study was carried out in strict accordance with the recommendations in the Guide for the Care and Use of Laboratory Animals of the National Institutes of Health, United States of America. The protocol was approved by The University of Georgia Institutional Animal Care and Use Committee (IACUC) (UGA Animal Use Protocol A2010-6-094), which oversees and provides veterinary care for all campus animal care facilities. IACUC is accredited by the Association for Assessment and Accreditation of Laboratory Animal Care International (AAALAC), is licensed by the US Department of Agriculture, and maintains an Assurance of Compliance with the US Public Health Service. IACUC technicians carried out all animal husbandry under strict guidelines to insure careful and consistent handling of animals.

### Mosquitoes

The UGAL strain of *A. aegypti* was used in all experiments. All life stages were maintained under a 12 h light/dark cycle at 28°C and 70–80% humidity. Larvae were fed with fish food, and adults were maintained in cages provided with 10% sucrose *ad libitum*.

### Endocrine reagents

ILP3 used in the study was synthesized and purified as previously described [2], while 20-hydroxyecdysone (20E) was purchased from Sigma.

### Hemocyte collection

Hemocytes were collected using the high injection/recovery method of Castillo et al. [15]. In brief, adult females were placed on ice for 15 min followed by injection of 3–6 µl of collection buffer (60% Schneider's medium (Sigma), 10% fetal bovine serum (FBS) (Hyclone) and 30% citrate buffer ( = anticoagulant) (98 mM NaOH, 186 mM NaCI, 1.7 mM EDTA and 41 mM citric acid, buffer pH 4.5) (vol/vol)) between the last two abdominal sclerites using a glass needle mounted on a micromanipulator. After 20 min 25 µl of collection buffer was injected into the lateral wall of the mesothorax and while concurrently collecting the diluted hemolymph by capillary action from the original injection site in the abdomen using a second hand-held glass needle.

### RT-PCR and rqRT-PCR analyses

Total RNA was extracted from hemocytes using Trizol reagent (Invitrogen). One µg of DNase-treated total RNA was used for cDNA synthesis using the Superscript III cDNA synthesis kit (Invitrogen). Hemocyte cDNA was diluted 10 fold and used 1 µl/20 µl for RT-PCR or 1 µl/10 µl for rqRT-PCR reactions with reaction mixtures containing specific primers for the *AaMIR* (forward 5′ CATT CCCGCTGAGCTACTTC 3′; reverse CGTCAGCACCTTCCTTCTTC 3′) or actin (control) (forward 5′ACCAACTGGGATGATATGGAG3′; reverse 5′ GTAGACAGTTTCGTGGATACCGCA3′) 0.2 mM of dNTPs and 2.5 Units Hotmaster Taq DNA polymerase (5 Prime GmbH, Hamburg, Germany) in 50-µl reaction mixtures. Cycling conditions were: 2 min of initial denaturation at 94°C, followed by 35 cycles of 20 sec denaturation step at 94°C, 30 sec annealing at 57°C, 45 sec extension at 72°C, and a final extension at 70°C for 10 min. Products were visualized on 0.5% agarose gels stained with ethidium bromide. Reactions for rqRT-PCR included iQ SYBR Green Supermix (BioRad; 5 µl/tube) and were run on a Rotor Gene Real Time-PCR cycler (Corbett) for 40 or 45 cycles under the following conditions: denaturation at 95° for 20 sec, annealing at 56° for 30 sec and extension at 72° for 30 sec. All reactions were run in quadruplicate with actin serving as the internal control (forward 5′ACCGCCG TCTACGATGCCA 3′; reverse 5′ATGGTGGTCTGCTGGTTCTT 3′). Data were analyzed by the ΔΔCt method as previously outlined [6].

### In situ hybridization

A 256 bp region of the *AaMIR* was PCR-amplified using the primers 5′ CCCCGGTTATGAAACAGTTC3′ (forward) and 5′TCTCGATGGACAAACTTCTT3′ reverse and cloned into the TOPO (pCR4.0) TA cloning dual promoter vector (Invitrogen, Carlsbad, CA). Plasmid was then digested with *Not*I and *Kpn*I (New England Biolabs), phenol/chloroform extracted and alcohol precipitated. Linearized plasmid was then used as template for synthesis of digoxigenin-labeled anti-sense and sense (negative control) *AaMIR* RNA probes using the DIG RNA labeling kit (SP6/T7) (Roche) following the manufacture's instructions. Probes were ethanol precipitated, resuspended in 25 µl of nuclease-free water and quantified by dot blot. In situ hybridization was then performed essentially as described by Lavine and Strand [25] using hemocytes from 1 day old females. Cells were placed in 96 well plates, allowed to settle for 30 min and fixed 4% para-formaldehyde in DEPC-treated Phosphate Buffer Saline (PBS) at 4°C for 20 minutes. After permeabilization with PBT (PBS+0.1% tween-20) for 15 min, cells were treated with Proteinase K digestion (0.1 µg/ml) for 90 sec. After refixation and washing with PBS, cells were incubated in 0.1 M Triethanolamine (TEA) buffer (pH 8.0) for 5 min, prehybridized in prehybridization buffer (2X SSC, 50% deionized formamide (Sigma), 10% dextran sulfate (Sigma) and 0.1 mg/ml Salmon sperm (Ambion)) for 1 hour at 55°C, and then hybridized for 24 h at 55° in hybridization buffer containing 100 ng/ml of probe. Following washing to high stringency for 1 h in 0.2X SSC at 55°C, cells were incubated with an anti-digoxigenin Fab fragment conjugated to alkaline phosphatase (Roche) in blocking solution (1∶5000) for 2 h at 37°C. Signal was visualized using NBT/BCIP in detection buffer followed by stoppage of reactions using TEA buffer (pH 8.0). Fixed hemocytes were also labeled with Alexa fluor 488 phalloidin and an anti-histone H1 antibody visualized using an Alexa fluor 546 secondary antibody as previously described [15]. All images were collected using a Leica inverted microscope and digital camera followed by export to Adobe Photoshop CS3 (Adobe) for assembly of figures.

### RNA interference (RNAi) assays

dsMIR and dsEGFP RNAs were produced using the T7 High Yield Transcription kit (Fermentas) [3]. dsRNAs were thereafter treated with DNAse1 and RNAseA, precipitated, and resuspended in nuclease-free water at 4 µg/µl for storage at −20°C. Mosquitoes were injected with 2 µg of dsRNA at 12 to16 h post-eclosion followed by collection of hemocytes for rqRT-PCR analysis as described above. Following natural log transformation of the 2^-ΔΔCT^ values, differences in transcript abundance value between the dsMIR and dsEGFP treatments were analyzed by t-test.

### Bioassays

The effects of decapitation on hemocyte abundance were conducted by decapitating females 1–12 h pbm followed by counting the total number of hemocytes recovered from females at 24 h pbm. The effects of ILP3 or 20E on hemocyte numbers at 24 h pbm were determined by decapitating females after feeding, injecting them with 0.5–50.0 pmol of ILP3 or 0.1–1.0 µg of 20E in 1 µl of PBS. Mosquitoes injected with PBS only served as a negative control. Decapitated females heal endogenously and visibly lose no hemolymph. Hemocyte abundance per mosquito was analyzed by ANOVA followed by the Tukey-Kramer multiple comparison procedure with treatment and/or sample time serving as the independent variable.

Bromodioxyuridine (BrdU) incorporation was assessed by feeding day 2 females a sugar solution containing 1 mg BrdU per ml (Sigma). BrdU-fed females were then blood fed on day 4, and either left intact or decapitated. Hemocyte samples were then collected 24 h pbm and processed [26]. Briefly, hemocytes were placed on L-lysine coated slides and incubated in Schneider's medium for 40 min, followed by fixation with paraformaldehyde and permeabilization using PBT. Cells were incubated in 2 N HCl for 40 min and neutralized in 0.1 M sodium borate (pH 8.5) followed by incubation in blocking reagent (Roche) for 1 h and a murine anti-BrdU antibody (1∶100; Invitrogen) overnight at 4°C. After washing in PBT and incubation with an Alexa Fluor 488-conjugated goat anti-mouse secondary antibody (1∶2000; Invitrogen) for 2 h, cells were examined and photographed as described above. To compare the percentage of BrdU-labeled hemocytes between intact and decapitated females, data were arcsin square root transformed and analyzed by ANOVA.

For infection assays, one cohort of adult females was blood fed (BF) on day 4 while a second cohort was left non-blood fed (NBF) followed 24 h later by injection of 1×10^3^–1×10^5^ colony forming units (CFUs) of the Migula strain of *E. coli* (ATCC# 25254), which are streptomycin-resistant. Third and fourth cohorts of mosquitoes were injected with dsEGFP or dsMIR RNA on day 1 followed by blood feeding on day 4 and injection with bacteria 24 h later as described above (BF-dsMIR). Mosquitoes were homogenized at 0, 3 or 24 h post-infection in 100 µl of BHI medium containing 50 µg/ml streptomycin, serially diluted and placed on BHI-streptomycin plates at 37°C for 18 h. The number of CFUs per individual was then determined (n = 5 females per treatment (BF, NBF, BF-dsEGFP, or BF-dsMIR), infection dose (1×10^3^–1×10^5^), and sample time (0, 3, 24 h) for a total of 180 individuals. We then compared the number of surviving bacteria per mosquito treatment and infection dose by Kruskal-Wallis tests. Other cohorts of BF and NBF mosquitoes treated as described above were maintained in cages (20 females per cage) with sugar water for 6 days post-infection. The number of mosquitoes surviving each day was then determined by counting the number of mosquitoes that died during the intervening 24 h period. The survival experiments were replicated three times using cohorts of 20 females per treatment and infection dose. To assess the effect of infection on mortality rates, we used the proportional hazards model where treatment and infection dose were the independent variables and the number of dead mosquitoes on each day was the dependent variable. All analyses were conducted using the JMP 7.0 statistical platform (SAS, Cary, NC).

## Results

### Granulocytes and oenocytoids express the AaMIR

**Figure 1:**
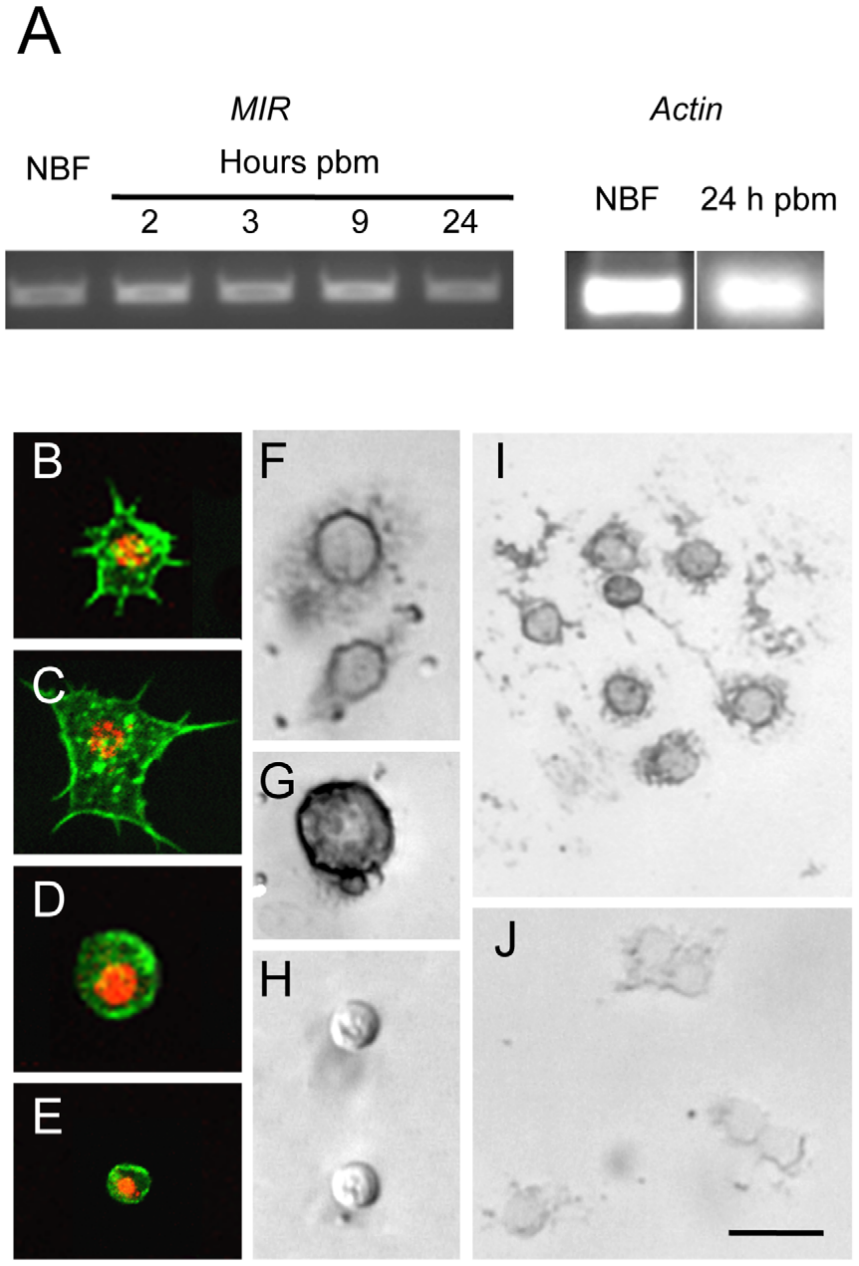
The *AaMIR* is expressed in granulocytes and oenocytoids of *A. aegypti*. (A) Ethidium bromide stained agarose gel showing the amplified *AaMIR* products from RT-PCR reactions using hemocyte cDNA templates from NBF females and BF females processed at 2, 3, 9 and 24 h pbm. Amplification of *A. aegypti* actin from NBF females and BF females at 24 h pbm served as the loading control. (B–E) *A. aegypti* hemocytes whose cytoplasms are labeled with phalloidin and whose nuclei are labeled using an anti-histone H1 antibody. B and C show representative granulocytes with numerous filopodia extending from each cell that strongly adhere each cell to the surface of a culture plate. D shows a representative oenocytoid, and E shows a representative prohemocyte. (F–I) In situ hybridization images of *A. aegypti* hemocytes hybridized with an *AaMIR* antisense probe. Strong hybridization signals are detected in the cytoplasm of granulocytes (F) and oenocytoids (G) but no signal is detected in prohemocytes (H). (I) Multiple granulocytes show a positive hybridization signal. (J) In situ hybridization image of *A. aegypti* hemocytes hybridized with an *AaMIR* sense probe. Note the absence of any hybridization signal. Scale bar in J equals 50 µm with images in the other panels being at a similar magnification.

*A. aegypti* encodes a single receptor tyrosine kinase, designated the mosquito insulin receptor (*AaMIR*), that is structurally homologous to the vertebrate IR [27] along with 8 ILP genes of which five (ILP1, 3, 4, 7, 8) are specifically expressed in medial neurosecretory cells in the brain [2], [28]. We previously determined that ILP3 dose-dependently stimulates yolk deposition into follicles, and that ILP3-induced egg development fully depends upon expression of the MIR in the ovaries [2], [3]. Given the similarities in timing between egg development and the increase in hemocyte abundance that occurs after blood feeding, we first assessed whether hemocytes also express the *AaMIR*. Using gene specific primers and total hemocyte cDNA as template, RT-PCR analysis detected *AaMIR* expression in hemocytes from both non-blood fed (NBF) females and blood-fed (BF) females sampled 2, 3, 9, and 24 h pbm (Fig. 1A). Granulocytes, oenocytoids, and prohemocytes are distinguished from one another in primary culture by their differences in size, biological activity, and morphology [15]. As shown in Fig. 1B and C, granulocytes rapidly attach to culture plates and extend filopodia of varying length. Oenocytoids in contrast remain spherical with a large prominent nucleus (Fig. 1D). Prohemocytes are also spherical but are distinctly smaller than oenocytoids (Fig. 1E). In *situ* hybridization analysis using an anti-sense *AaMIR* probe detected strong hybridization signals in the cytoplasm of virtually all granulocytes and oenocytoids from NBF and BF females, but detected no signal in prohemocytes (Fig. 1F–I). We also detected no signal in any hemocyte type using a sense *AaMIR* probe (Fig. 1J).

### Decapitation blocks the increase in hemocyte abundance that occurs after blood feeding

**Figure 2:**
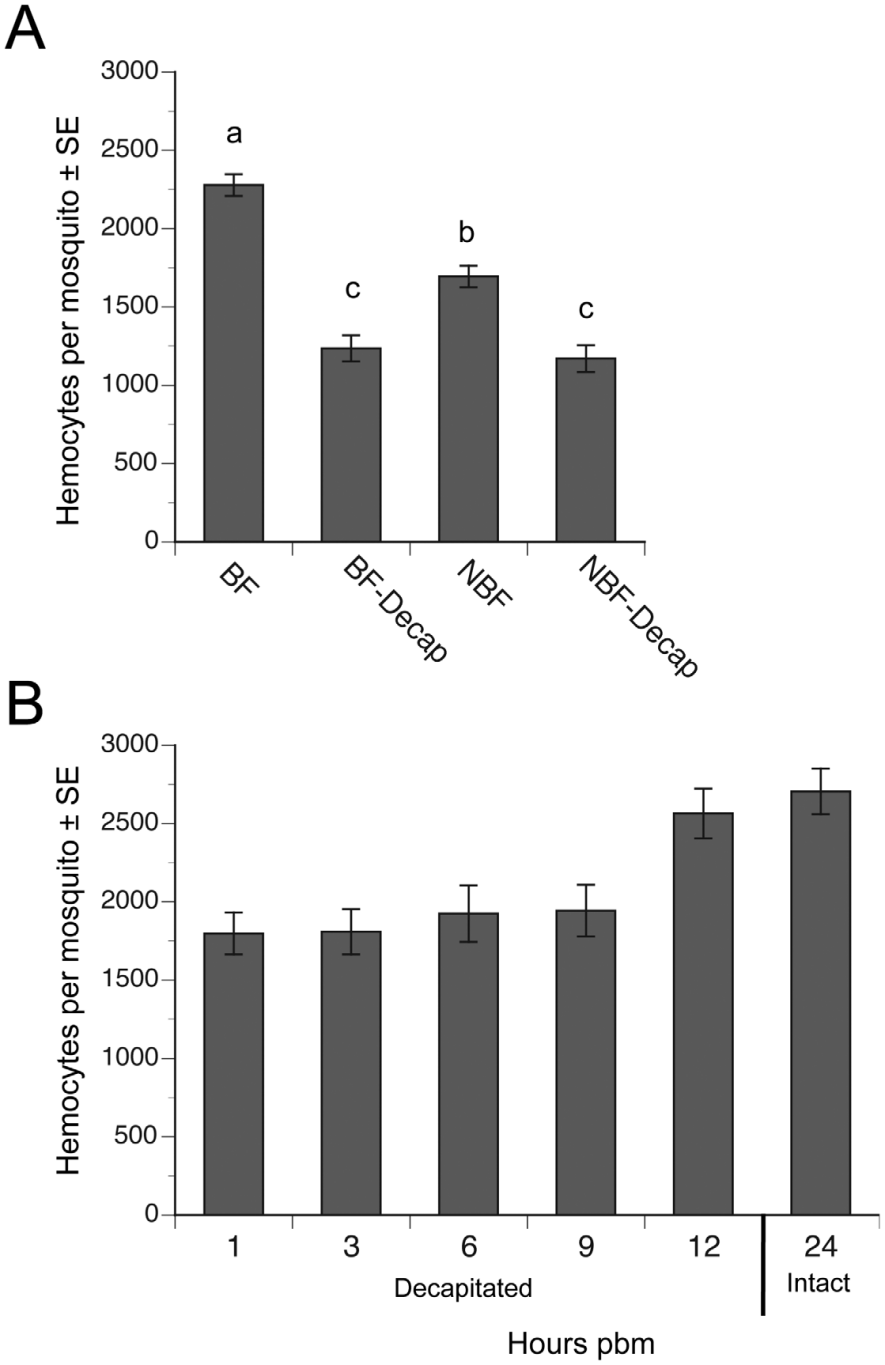
Decapitation inhibits the increase in hemocyte abundance that occurs after blood feeding. (A) Mean number (± SE) of hemocytes per mosquito in BF females, BF females decapitated 2 h pbm (BF-Decap), NBF females, and NBF-Decap females. Adult females were blood fed four days post-emergence and hemocyte numbers were determined 24 h later (day 5). NBF females were also decapitated on day four and examined on day 5. Different letters above a given bar indicate means that significantly differ (F_3, 142_ = 45.7; P<0.0001). (B) Mean number (± SE) of hemocytes per day 4 mosquito decapitated 1, 3, 6, 9 or 12 h pbm. All females were bled at 24 h pbm and compared to the number of hemocytes collected from intact (non-decapitated) females that were also bled at 24 h pbm (control). Females decapitated 1–9 h pbm contained significantly fewer hemocytes than non-decapitated control females, but females decapitated at 12 h pbm contained the same number of hemocytes as controls (asterisks) (F_5, 72_ = 6.7; P<0.0001).

Since decapitation of females after blood feeding blocks yolk deposition into eggs [2], [3], we asked if decapitation also blocked the increase in hemocytes that occurs after blood feeding. Consistent with earlier studies [15], [17], we recovered significantly more hemocytes from BF females at 24 h pbm than from NBF females of the same age (Fig. 2A). This increase resulted in BF females on average containing 43% more hemocytes than NBF females. In contrast, decapitation of BF females at 2 h pbm fully blocked this increase in abundance (Fig. 2A). Decapitation of NBF females also resulted in a small but significant reduction in the total number of hemocytes recovered relative to intact (non-decapidated) NBF females at 24 h (Fig. 2A). Time course studies indicated that decapitation of BF females up to 9 h pbm prevented hemocyte numbers from increasing to the same level as non-decapitated BF females at 24 h pbm (Fig. 2B). However, females decapitated at 12 h pbm contained the same number of hemocytes as intact BF females at 24 h (Fig. 2B).

### A higher proportion of hemocytes incorporate BrdU following blood feeding

**Figure 3:**
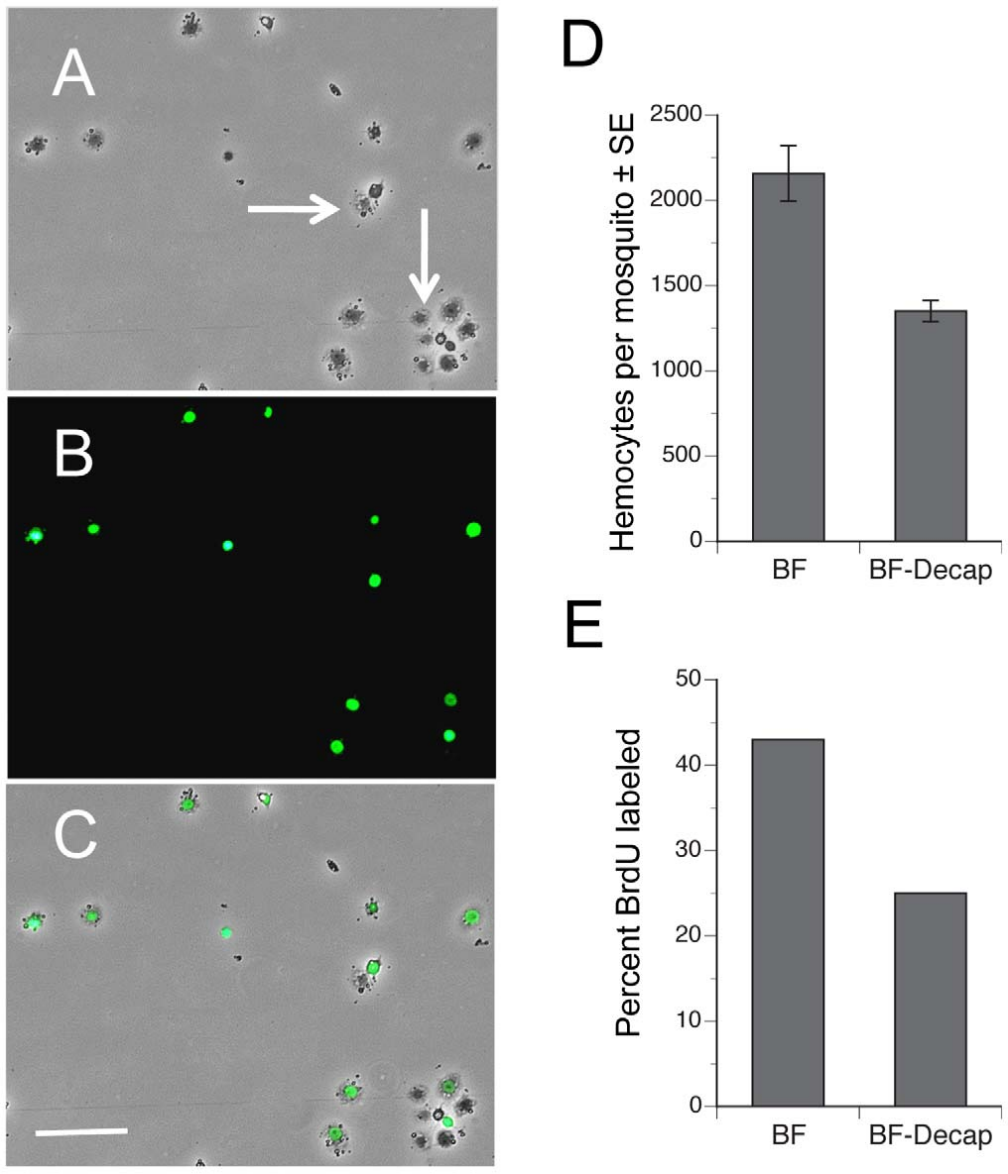
Blood feeding increases the proportion of hemocytes labeled by BrdU. Phase-contrast (A), epiflorescent (B) and a phase-epifluorescent merged image (C) of *A. aegypti* hemocytes from a BF female collected 24 h pbm. The nuclei of actively cycling hemocytes that incorporated BrdU are labeled green, whereas the nuclei of representative unlabeled hemocytes exhibit no nuclear signal (arrows). Note the spread morphology of the labeled cells indicating their identity as granulocytes. Scale bar in C equals 100 µm. (D) BF intact females treated with BrdU contain more hemocytes (± SE) than females decapitated immediately after blood feeding (F_1, 18_ = 8.4; P<0.001). Females were pretreated with BrdU and blood fed on day 4. Hemocytes were then collected 24 h pbm. (E) Intact BF females treated with BrdU contain a higher proportion of BrdU-labeled hemocytes than females decapitated immediately after blood feeding (F_1, 18_ = 21.5; P<0.0002).

The increase in the number of hemocytes recovered from females after blood feeding could reflect either the entry of previously sessile hemocytes into circulation, an increase in the proportion of actively cycling hemocytes, or both. If events associated with blood feeding increase the number of actively cycling hemocytes, we reasoned that the proportion of hemocytes incorporating an exogenous DNA precursor like BrdU during S phase of the cell cycle should increase. We also reasoned *A. aegypti* should uptake BrdU by feeding since this approach has been successfully used to BrdU label proliferating cells in other insects [29]–[31]. We therefore fed adult females sugar water containing BrdU and then compared the proportion of BrdU-labeled hemocytes in intact females 24 h pbm to females that we decapitated immediately after blood feeding. Examination of hemocytes by epifluorescence microscopy showed that feeding of BrdU resulted in strong nuclear labeling of some but not all hemocytes recovered from each female (Fig. 3A–C). As with earlier experiments, we also recovered significantly more hemocytes from intact than decapitated BF females, which indicated that BrdU feeding did not alter the increase in hemocyte abundance that normally occurs after a blood meal (Fig. 3D). However, when we assessed the percentage of hemocytes with incorporated BrdU, our results showed that significantly more hemocytes from intact, BF females were BrdU-labeled than from decapitated, BF females, which suggested that blood feeding increased the proportion of actively cycling hemocytes (Fig. 3E). Based upon morphology, we further concluded that virtually all BrdU-labeled hemocytes (>99%) were granulocytes.

### ILP3 dose-dependently restores hemocyte abundance in blood-fed, decapitated females

**Figure 4:**
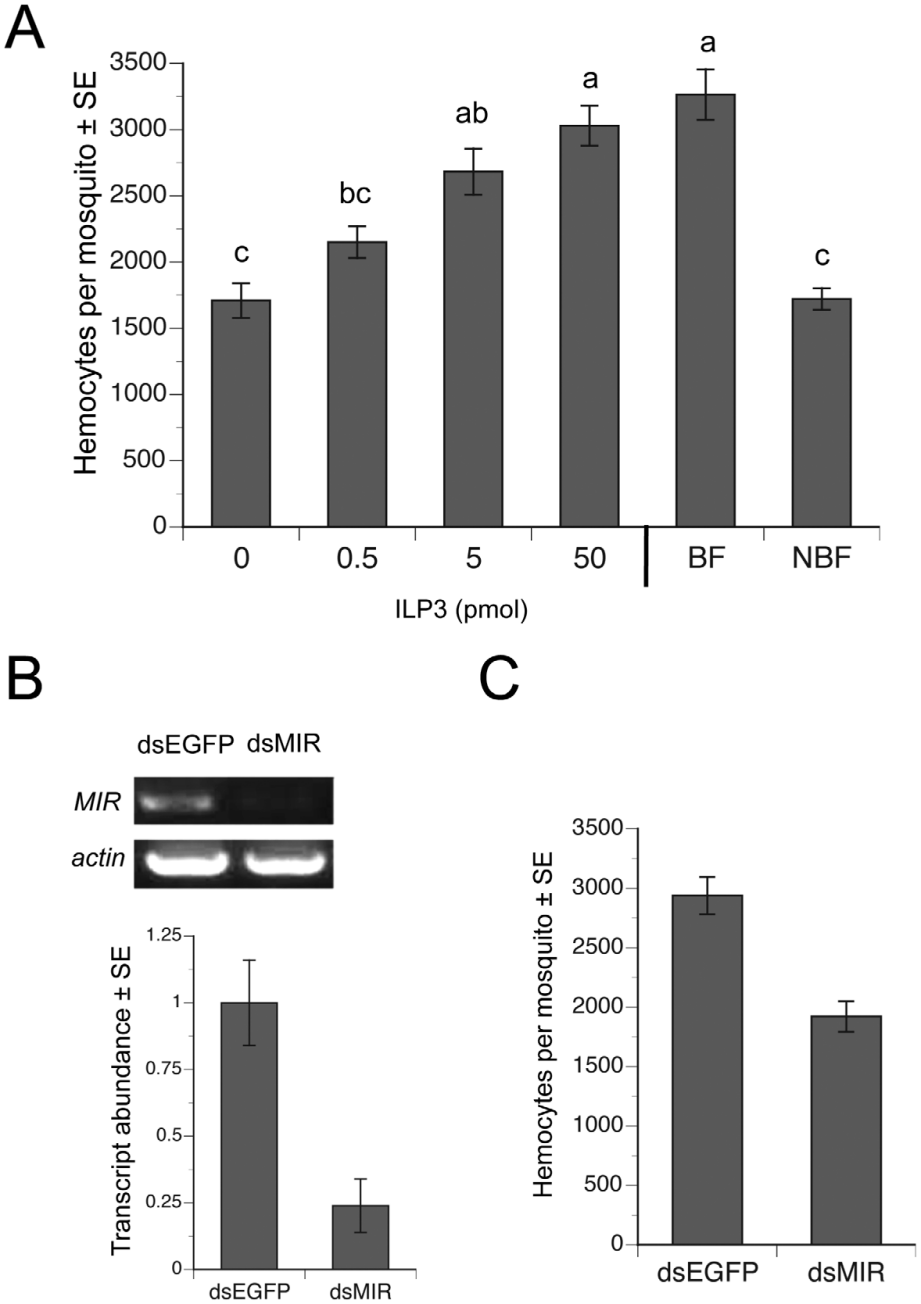
ILP3 dose-dependently rescues hemocyte abundance after decapitation while dsMIR treatment reduces hemocyte abundance. (A) Day four females were blood fed, decapitated and injected with a single dose of 0.5–50 pmol of ILP3. BF-decapitated females injected with water only (0) and intact, NBF females served as negative controls, while intact BF females (BF) served as a positive control. Mean number (± SE) of hemocytes for all treatments was determined at 24 h pbm. Different letters above a given bar indicate means that significantly differ (F_5, 60_ = 20.9; P<0.0001). (B) dsMIR RNA reduces *AaMIR* transcript abundance in hemocytes. The top of the figure shows RT-PCR products for the *MIR* relative to actin in hemocyte samples from mosquitoes treated with dsEGFP (control) or dsMIR RNA at 1 day post-eclosion. Hemocyte RNA pooled from 10 day 4 BF females 24 h pbm served as the source of template. The bottom of the figure shows rqRT-PCR analysis of *MIR* transcript abundance (± SE) in mosquito hemocytes 24 h pbm pretreated with dsEGFP or dsMIR. Transcript levels were standardized to a level of 1 for hemocytes from dsEGFP females, while transcript levels for dsMIR-treated females are expressed relative to the control. Each treatment was replicated three times using samples from 10 pooled females. dsMIR treatment significantly reduced *MIR* transcript abundance relative to the dsEGFP control (t-test, P<0.05). (C) dsMIR treatment reduced the mean number of hemocytes (± SE) per female relative to females treated with dsEGFP (F_1, 34_ = 30.7; P<0.0001).

Since 5 pmol or more of ILP3 rescues egg development in decapitated, BF females [2], we injected females with a single dose of ILP3 that ranged from 0.5–50 pmol. Our results showed that a 50 pmol injection fully restored hemocyte numbers in decapitated BF females to the same level found in intact BF females, while lesser amounts of ILP3 resulted in lower levels of hemocyte recovery (Fig. 4A). Decapitated, BF females injected with a range of concentrations of 20E in contrast exhibited no increases in hemocyte abundance (data not presented). To assess whether this rescue effect depended upon *AaMIR* expression, we injected dsMIR or dsEGFP (negative control) RNA into newly eclosed females, held them for 4 days with water, and then blood fed on day 4. rqRT-PCR analysis confirmed that dsMIR treatment reduced *AaMIR* transcript abundance in hemocytes relative to mosquitoes treated with dsEGFP (Fig. 4B), while bioassay results showed that dsMIR-treated females contained significantly fewer hemocytes than females pretreated with dsEGFP (Fig. 4C).

### Increased hemocyte abundance enhances clearance of E. coli at lower levels of initial infection

**Figure 5:**
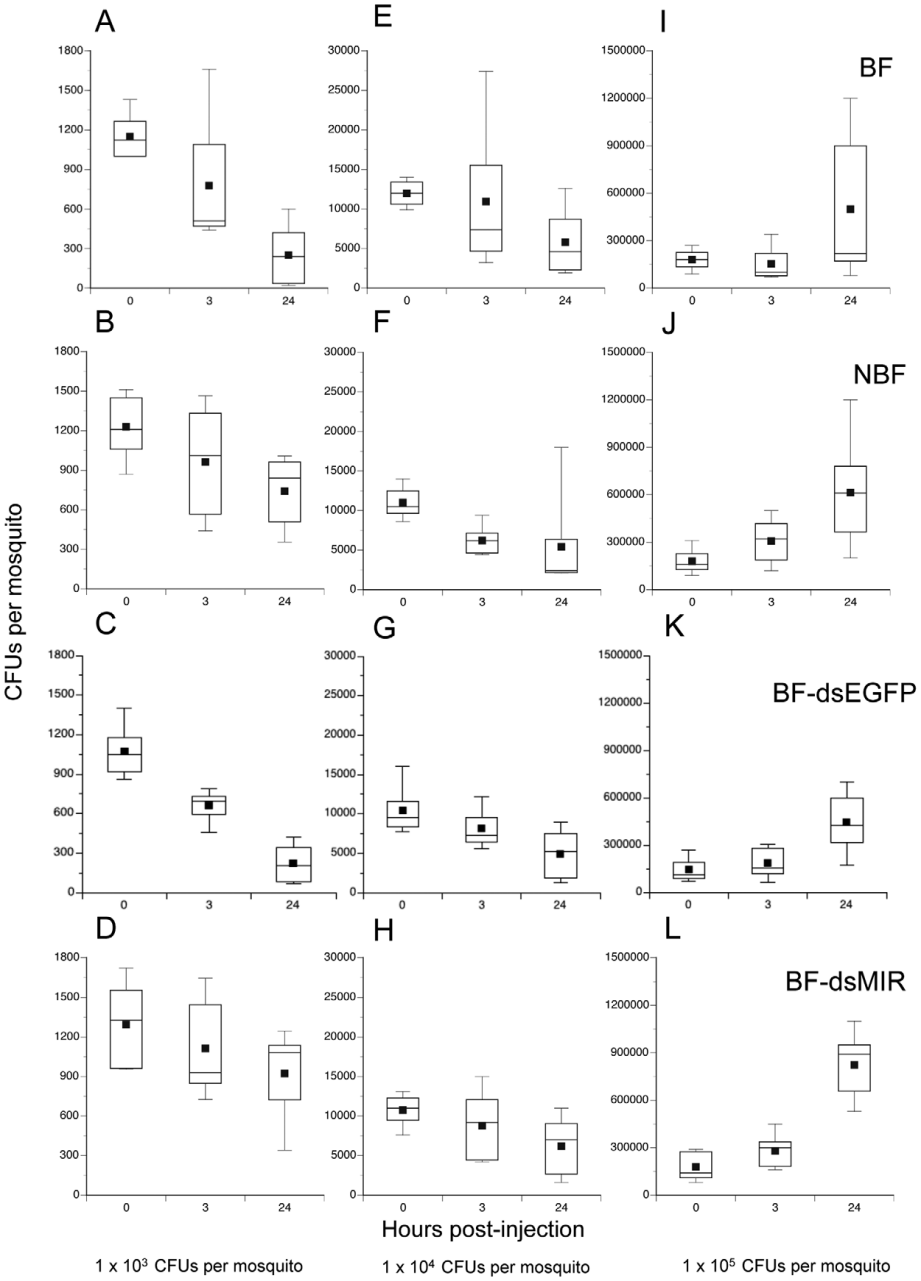
Survival of *E. coli* in *A. aegypti* varies with feeding and initial infection dose. BF females (first row), NBF females (second row), BF females pretreated with dsEGFP (BF-dsEGFP) (third row), and BF females pretreated with dsMIR RNA (BF-dsMIR) (fourth row) were fed on day 4, and then infected 24 h pbm (day 5) with 1×10^3^ (left column), 1×10^4^ (middle column), or 1×10^5^ (right column) CFUs of *E. coli*. Females from each treatment and infection dose were then processed to determine the CFUs of surviving bacteria at 0, 3 or 24 h post-infection. (A) BF females infected with 1×10^3^ CFUs of *E. coli*. (B) NBF females infected with 1×10^3^ CFUs of *E. coli*. (C) BF-dsEGFP females infected with 1×10^3^ CFUs of *E. coli*. (D) BF-dsMIR females infected with 1×10^3^ CFUs of *E. coli*. (E) BF females infected with 1×10^4^ CFUs of *E. coli*. (F) NBF females infected with 1×10^4^ CFUs of *E. coli*. (G) BF-dsEGFP females infected with 1×10^4^ CFUs of *E. coli*. (H) BF-dsMIR females infected with 1×10^4^ CFUs of *E. coli*. (I) BF-dsMIR females infected with 1×10^5^ CFUs of *E. coli*. (J) NBF females treated with 1×10^5^ CFUs of *E. coli*. (K) BF-dsEGFP females treated with 1×10^5^ CFUs of *E. coli*. (L) BF-dsMIR females treated with 1×10^5^ CFUs of *E. coli*. Each graph shows a stem and leaf plot with the lower and upper quartiles indicated by the large box. Mean survival is indicated by the small black square in the box, a horizontal bar indicates the median, and vertical lines extending from each box indicate the most extreme values.

Given previous evidence showing that 1) granulocytes phagocytize several species of bacteria including *Escherichia coli* [13], [15], and 2) age-dependent reductions in hemocyte abundance correlate with increased susceptibility to *E. coli* infection [13], we asked whether increases in hemocyte abundance following blood feeding enhances clearance of bacteria. One cohort of females was pretreated with dsMIR RNA while another was treated with dsEGFP which served as both an RNAi and wounding control. Two other cohorts were left untreated. We blood fed on day 4 the dsMIR and dsEGFP-treated cohorts and one of the untreated cohorts followed 24 h later by infection of mosquitoes in each treatment with three different amounts of streptomycin-resistant *E. coli* (1×10^3^, 10^4^ or 10^5^ colony forming units (CFUs). This resulted in the following treatments: BF, NBF, BF-dsEGFP injected, and BF-dsMIR injected. We then determined the number of surviving bacteria at 0, 3, and 24 h post-injection by homogenizing each mosquito and culturing the supernatant on streptomycin-containing BHI plates. Overall, the number of bacteria recovered per mosquito varied significantly among treatments, infection dose, and sample time (Kruskal-Wallis test: X^2^ = 166.3; df = 35; P = 0.0001) (Fig. 5). Focusing on the lowest infection dose (1×10^3^ CFUs) and 24 h sampling time, our results indicated that bacterial survival varied significantly among treatments (X^2^ = 11.0; df = 3; P = 0.012) (Fig. 5A–D). Closer inspection of these data showed that clearance of bacteria by BF and BF-dsEGFP females did not differ from one another (X^2^ = 0.01; df = 1; P = 0.9) while clearance of bacteria by NBF and BF-dsMIR females also did not differ (X^2^ = 0.03; df = 1; P = 0.8). In contrast, BF and BF-dsEGFP females cleared significantly more bacteria than NBF and BF-dsMIR females (X^2^ = 10.6; df = 1; P = 0.0012). Taken together, these results argued that increased production of hemocytes by BF and BF-dsEGFP females enhanced clearance of bacteria while pretreatment with dsMIR reduced hemocyte production and associated clearance of bacteria by BF females to similar levels as NBF females. These data also suggested that wounding at the time of dsRNA injection had no effect on the outcome of these assays. At the intermediate infection dose we tested (1×10^4^ CFUs), the number of surviving bacteria after 24 h was also lower but no differences were detected among treatments (X^2^ = 0.68; df = 3; P = 0.88) (Fig. 5E–H), while at the highest dose (1×10^5^ CFUs) the number of surviving bacteria at 24 h had similarly increased in each treatment (X^2^ = 3.8; df = 3; P = 0.29) (Fig. 5I–L). Inspection of hemocytes from mosquitoes across all treatments and infection doses showed that most granulocytes contained numerous internalized bacteria at both 3 and 24 h post-infection (data not presented). The data presented in Fig. 5, however, also showed that no mosquitoes fully cleared the infection by 24 h at any of the three infection doses tested.

### Blood fed mosquitoes are more susceptible to *E. coli* at higher levels of infection

**Figure 6:**
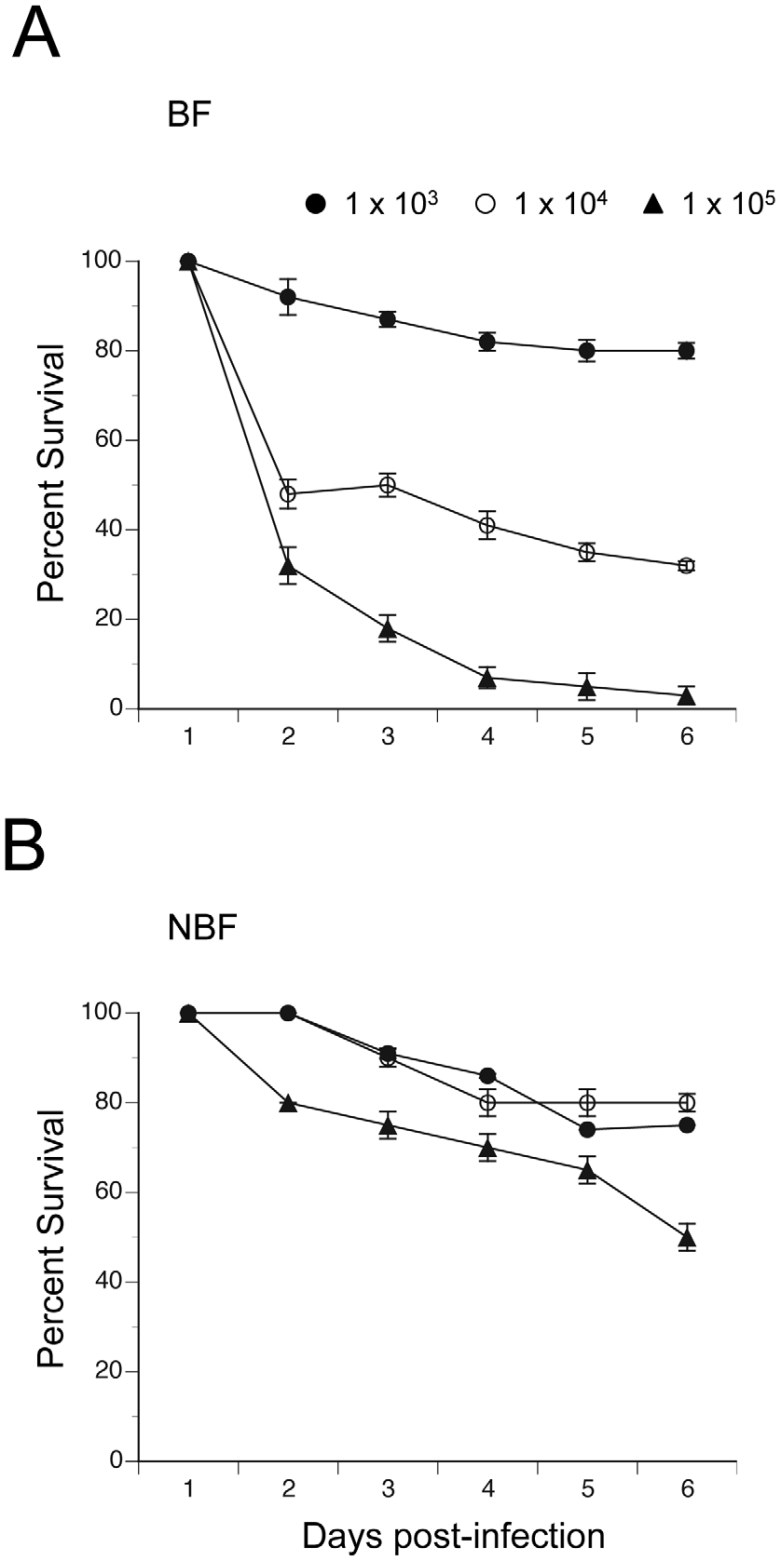
Survival curves (± 1 SE) for BF and NBF females fed on day 4 and infected 24 h pbm (day 5) with different doses of *E. coli*. (A) BF females infected with 1×10^3^, 1×10^4^ or 1×10^5^ CFUs of *E. coli*. (B) NBF females infected with 1×10^3^, 1×10^4^ or 1×10^5^ CFUs of *E. coli*.

We assessed the relationship between clearance of *E. coli* by BF and NBF females and survival by monitoring mosquito mortality for 6 days post-infection. Fig. 6 shows that nearly all BF and NBF females survived infection by 1×10^3^ CFUs. However, analysis across all three levels of infection using the proportional hazards model revealed a strongly significant difference in the death rate of BF and NBF females (X^2^ = 27.4; df = 3; P<0.0001) (Fig. 6). Effect likelihood ratio tests confirmed, as expected, that death rate increased with infection dose (X^2^ = 19.1; df = 1; P<0.0001), but also showed that BF females died at higher rates than NBF females (X^2^ = 11.3; df = 1; P<0.0001) (Fig. 6). Thus, few NBF females died after 6 days when infected with 1×10^4^ CFUs of *E. coli*, whereas the same dose resulted in more than 50% of BF females dying within 2 days of infection. Similarly, infection with 1×10^5^ CFUs resulted in only 20% mortality of NBF females after 2 days and 50% mortality after 6 days. In contrast, the same dose of *E. coli* killed 75% of BF females within 2 days and 95% of females after 6 (Fig. 6). Since most phagocytosis of *E. coli* occurs within the first 24 h of infection [13] and bacterial survival after 24 h positively correlates with initial infection dose (Fig. 5), the data presented in Fig. 6 suggested that differential survival of BF and NBF females reflected an asymmetric response to surviving bacteria that hemocytes failed to clear.

## Discussion

Most studies of the mosquito immune system focus on defense responses following systemic infection with bacteria or consumption of a pathogen-infected blood meal [11], [17], [32], [33]. In *A. aegypti*, for example, bacterial infection of the hemocoel elicits a phagocytic response by hemocytes followed by inducible expression of antimicrobial peptides [13], [21], while entry into the hemocoel from the gut by filarial nematodes activates cellular and melanotic defenses [34]. In the malaria vector *Anopheles gambiae*, passage of *Plasmodium* to the hemolymph-bathed basal side of the midgut exposes the parasite to complement-like thioester-containing proteins from hemocytes that contribute to parasite killing [18]. *Plasmodium* infection also damages the peritrophic matrix that surrounds the blood meal, resulting in commensal bacteria interacting with the midgut, which in turn stimulates constitutive and inducible defense responses against both bacteria and *Plasmodium* [24], [35]–[37].

Far less is known about how mosquitoes invest in immune defenses in the absence of infection. We reasoned that the unique requirement of vector species to blood feed also creates a cyclical pattern of increased nutrient availability and infection risk that could favor cyclical investment in constitutive defenses like hemocytes. Our results support this hypothesis by showing that the number of hemocytes recovered from females increases 40% by 24 pbm. Our results further reveal a previously unrecognized role for the insulin signaling pathway in regulating the cellular arm of the mosquito immune system and synchronizing increased production of hemocytes with reproduction.

The progressive, age-dependent decline in hemocyte abundance that occurs in the absence of blood feeding suggested to Hillyer and coworkers [13] that hemocytes do not proliferate in adult mosquitoes. However, our BrdU labeling experiments indicate that actively cycling hemocytes exist in both NBF and BF females, while the significant rise in BrdU-labeled hemocytes following a blood meal strongly suggests that increases in hemocyte abundance are due at least in part to proliferation of phagocytic granulocytes. The longer term priming experiments of Rodriguez et al. [24] suggest that granulocytes also proliferate in adult female *A. gambiae*. However, the average number of hemocytes per individual we and others recover from *A. aegypti* and *A. gambiae* [13], [15] is much lower than the number of hemocytes per mosquito reported by Rodriguez et al. [24]. Entry of sessile hemocytes into circulation could also contribute to the increase in hemocyte abundance after blood feeding given evidence that mobilization of sessile hemocytes can elevate the abundance of circulating hemocytes in other insects [38]. Our use of an anticoagulant to collect hemocytes, however, reduces adhesion of mosquito hemocytes and likely enhances collection of both circulating and sessile cells. Thus, the increased numbers of hemocytes recovered from BF females is likely not due to NBF females containing more sessile hemocytes that we failed to recover.

Unlike mammals, which produce both insulin and insulin-like growth factors that preferentially interact with different receptors, *A. aegypti* and other insects encode only a single receptor tyrosine kinase (IR) that functions as the receptor for multiple ILP family members [2], [3]. Our own studies indicate that ILP3 exhibits both metabolic (insulin-like) and growth-promoting (insulin growth factor-like) activity in regard to regulating egg development [2], [3], [6]. Our finding during the current study that granulocytes and oenocytoids express the MIR is also consistent with the suggestion that mosquito hemocytes proliferate in direct response to ILP3. The ILP bombyxin has likewise been implicated in stimulating hemocyte proliferation in the silkmoth, *Bombyx mori* [39], while studies with mammals implicate insulin growth factor-like signaling in proliferation of lymphocytes [40]–[42]. A recent study also implicates a cell surface protein designated crustacean hematopoietic factor (CHF) in survival of certain hemocyte types from the crayfish *Pacifastacus leniusculus* [43]. Interestingly, CHF contains an insulin growth factor binding protein domain, but it remains unknown whether CHF interacts with any crustacean ILP [43]. The absence of MIR expression and failure to detect any BrdU labeling of prohemocytes suggests that the increases in granulocyte abundance that occur after blood feeding are likely due to proliferation of granulocytes themselves and not the differentiation of prohemocytes into granulocytes. It remains unclear whether prohemocytes proliferate during other life stages but overall the results of the current study do not provide any evidence that the circulating cells named prohemocytes in prior studies [15], [17], [24] have a progenitor function in adult *Ae. aegypti*.

We recognize our in vivo experiments leave open the possibility that the effects of ILP3 on hemocyte proliferation could be indirect. That is, ILP-stimulated nutrient acquisition following blood feeding could be responsible for increased proliferation of hemocytes. Unfortunately, *A. aegypti* hemocytes are labile in primary culture and we currently are unable to maintain them for a long enough period to evaluate whether ILP3 directly stimulates proliferation in vitro. However, we do note that injection of ILP3 (50 pmol) into NBF females stimulates a significant increase in hemocyte abundance relative to NBF controls after 24 h (data not presented). This increase is smaller than the increase observed in decapitated BF females (Fig. 4), but these results strongly suggest that ILP3-stimulated proliferation of hemocytes is not solely due to increased availability of nutrients from digestion of the blood meal.

In vertebrates, activation of nuclear hormone receptors by their steroid hormone ligands affects both adaptive and innate immune responses [40], [44], [45]. Much less is known about the role of steroid hormones in regulating the insect immune system although 20E does up-regulate expression of a prophenoloxidase gene in an *A. gambiae* cell line of hemocyte origin [46] and selected antimicrobial peptide genes in the S2 and mbn-2 cell lines from *Drosophila* [47], [48]. 20E also stimulates proliferation of mbn-2 cells and phagocytosis by *Drosophila* hemocytes in vivo [49], [50]. As previously noted, release of ILP3 from the brain after blood feeding strongly upregulates the synthesis and release of 20E and other ECDs by the ovaries [2], which then interact with ILPs and TOR signaling to regulate the enzymes that digest the blood meal and yolk protein expression by the fat body [6], [7]. However, results of the current study indicate that 20E has no stimulatory effect on proliferation of hemocytes in *A. aegypti*.

We anticipated that blood feeding would elevate resistance to at least some potential pathogens through an increase in the number of phagocytic granulocytes. We examined this possibility using *E. coli* because earlier studies showed that *A. aegypti* granulocytes readily phagocytize this bacterium [12], [15], [17], while also showing that *E. coli* elicits a dose-dependent mortality response [13]. Consistent with expectations, our results indicate that BF females clear more bacteria than NBF females at the lowest level of infection (10^3^ CFUs per female) we tested. Our results showing that pretreatment of BF females with dsMIR reduces bacterial clearance to NBF levels also supports the conclusion that enhanced clearance by BF females is due to increased numbers of granulocytes. No difference in clearance of bacteria was detected among treatments at higher levels of infection. However, this outcome was not entirely unexpected given that hemocyte abundance after blood feeding increases less than 2-fold but females were injected with 10 or 100-fold more bacteria. In effect, infection by 10^4^ or 10^5^ *E. coli* exceeded the phagocytic capacity of the granulocytes present in each treatment resulting in higher numbers of surviving bacteria after 24 h. Although Hillyer et al. [13] estimated that *A. aegypti* granulocytes can phagocytize more than 1000 *E. coli* per cell, they also detected levels of surviving bacteria 24 h and 5 days post-infection that are very similar to our results with NBF females suggesting substantial numbers of bacteria evaded phagocytosis in vivo when mosquitoes were infected with 10^4^ or more cells.

In contrast, we did not anticipate that NBF females would better survive intermediate and high levels of *E. coli* infection than BF females. When viewed together, the results presented in Fig. 5 and 6 indicate that blood feeding enhances resistance at the lowest level of infection we tested as measured by reduced survival of *E. coli*. However, at higher levels of initial infection blood feeding reduces tolerance to *E. coli* which is defined in the insect immunity literature as the ability to survive infection at a given pathogen load [51]. Thus, while hemocytes clear substantial numbers of bacteria in the early stages of infection, surviving bacteria adversely affect the survival of BF females more than NBF females. Resistance and tolerance in insects have previously been shown to vary with host genotype and pathogen species [52]–[54]. Our results add to this literature by showing that resistance and tolerance to the same pathogen dramatically changes in *A. aegypti* as a consequence of blood meal digestion and/or mobilization of resources for reproduction.

The mechanism(s) underlying reduced tolerance to *E. coli* in BF females remains unclear. In *Drosophila*, both bacterial infection and infection-independent activation of the Toll signaling pathway suppresses insulin signaling which reduces energy stores but enhances tolerance by potentially increasing investment in inducible defenses under Toll regulation [55], [56]. While blood feeding activates insulin signaling and other processes related to yolk protein production in *A. aegypti* [2], [7], [8], studies in *A. gambiae* indicate that the presence of Vg diminishes killing of *Plasmodium* [57]. Thus, our finding of diminished tolerance could reflect either the effects of bacterially induced NF-kB signaling, the effects of Vg interfering with killing of bacteria, or changes in allocation of nutritional resources that adversely impact tolerance. Interestingly, activation of NF-kB signaling following *Plasmodium* infection also reduces Vg expression in *A. gambiae* [57], which in light of the aforementioned studies with *Drosophila* could reflect an indirect response mediated by reduced insulin signaling. A second possibility for reduced tolerance is that metabolism of vertebrate blood is both energetically expensive and generates toxic compounds like heme or ammonia that must be inactivated [58]. Recent findings by Oliveira et al. [59], for example, show that blood meal-derived heme reduces production of reactive oxygen species (ROS) in the midgut of *A. aegypti*, which normally regulates growth of gut microbiota but upon down regulation allows the gut microbiota to greatly increase in abundance. It is unlikely reduced ROS production in the midgut affected the systemic infection of the hemocoel we generated in our experiments. However, these results do suggest other processes related to tolerance could be adversely affected by processing the blood meal.
